# Challenges navigating publicly funded home care in Ontario, Canada: Perspectives from unpaid caregivers of persons living with dementia

**DOI:** 10.1177/14713012231190579

**Published:** 2023-07-19

**Authors:** Husayn Marani, James Shaw, Gregory P Marchildon

**Affiliations:** Institute of Health Policy, Management and Evaluation, Dalla Lana School of Public Health, 206712 University of Toronto, Toronto, ON, Canada; North American Observatory on Health Systems and Policies, 206712 University of Toronto, Toronto, ON, Canada; Institute of Health Policy, Management and Evaluation, Dalla Lana School of Public Health, 206712 University of Toronto, Toronto, ON, Canada; Department of Physical Therapy, 7938 University of Toronto, Toronto, ON, Canada; Institute of Health Policy, Management and Evaluation, Dalla Lana School of Public Health, 206712 University of Toronto, Toronto, ON, Canada; North American Observatory on Health Systems and Policies, 206712 University of Toronto, Toronto, ON, Canada

**Keywords:** home care, financial risk, unpaid care, dementia

## Abstract

In Canada, persons living with dementia represent a sizable number of home care recipients. Although home care is not wholly publicly funded under provincial health insurance plans, some provinces like Ontario subsidize a maximum number of hours of home care provided by a personal support worker (PSW) on the basis of need. The public subsidization of home care may be interpreted as a mechanism of financial risk protection, enabling unpaid caregivers to maintain employment, income levels, and personal health. However, deficits in the availability of home care call into question how the organization of home care may be contributing to financial risk among unpaid caregivers. Inspired by qualitative phenomenology, this study describes the financial risks experienced by unpaid caregivers of persons living with dementia navigating publicly funded homecare in Ontario. Based on 24 interviews conducted between August-December, 2020, we found financial risk emerges across three dimensions: 1) receiving information about publicly funded home care that anticipates future care needs; 2) receiving flexible hours of support from a PSW; and 3) maintaining consistent access to quality support. Financial risks included turning to privately funded home care options, or taking time off work to provide care. Findings may inform local and international home care reforms aiming to protect caregivers from financial risk.

## Introduction

Globally, the prevalence of dementia correlated with an aging population is expected to rise. This has important economic implications. In the Canadian context, the cost attributable to Canada’s aging population is six times greater for dependent persons living with dementia than the cost for the same dementia-free demographic (Chambers et al., 2016). In an effort to curb the economic burden of dementia on historically costly sectors of the health system, including acute care settings such as hospitals and social care settings, including the home and residential long-term care facilities, jurisdictions across Canada have made significant strides in improving health service delivery at home. Although home care is not included as an “insured service” under the *Canada Health Act* (1984) and therefore, is not part of universal health coverage in Canada, provincial governments do subsidize a maximum number of hours of home care provided by home care workers on a needs-tested basis, which we further describe below in the context of home care. This is fairly consistent with Canada’s liberal stance of social benefit distribution (Spicker, 2013). However, changes to regulations around home and community care services are underway in Ontario to remove service maximums (Government of Ontario, 2022a).

Of the 1.1 million Canadians (2.8% of the population) expected to be living with dementia by 2038, 40% will be living in Ontario (Government of Ontario, 2016), 63% of whom are expected to be living at home. Despite this trend, interventions that have emerged from Ontario’s own Aging-at-Home strategy (Government of Ontario, 2010), namely improving public funding for home care workers, appear to have resulted in gaps in service availability proving to be uniquely problematic for persons living with dementia. In Ontario, home care workers called personal support workers (PSWs) support persons living with dementia with activities of daily living, including dressing, bathing and medication compliance; however, the number of publicly funded PSW hours is insufficient for persons living with dementia, who often need continuous care and monitoring (Marani, McKay, & Marchildon, 2021). In addition, PSWs may be unable to help persons living with dementia who cannot explain their needs constantly (DaSilva, 2015; Laucius, 2018). These conditions have imposed a significant care and financial responsibility on their unpaid caregivers (e.g., family members, friends or neighbours) who, consequently, may need to maintain a consistent role in the daily provision of care for their care recipient (Lilly et al., 2012; Ontario Caregiver Organization [OCO], 2020). Indeed, there is evidence emerging that gaps in the supply of publicly funded home care provided by a PSW have been filled by unpaid caregivers who have taken on more weekly hours providing unpaid care (Canadian Institute for Health Information [CIHI], 2020). These gaps may have been exacerbated by the COVID-19 pandemic, during which caregivers in Ontario have noted a decrease in the availability of publicly funded home care provided by a PSW (Marani et al., 2023).

There is growing research and policy interest in the implications of these gaps in service availability on caregivers’ financial well-being. These invisible costs may be contributing to financial insecurity among caregivers, which could conflict with Ontario’s historically liberal stance on social benefit distribution (Olsen, 1994). Evidence suggests that, by 2038, the annual loss in production as a proportion of GDP attributed to persons living with dementia and their caregivers is estimated to be close to $6.7 billion CAD (Smetanin et al., 2009). Accordingly, we suggest that the public subsidization of PSWs is an important mechanism of financial risk protection— defined as public protection from both the magnitude of out-of-pocket care expenditure, and the impacts of these expenses across domains of financial risk, including income-generating potential, employment productivity, and overall personal health (Marani et al., 2023). Specifically, subsidizing home care could maintain caregiver productivity through formal employment (McKee et al., 2013). While estimates of productivity losses among caregivers of persons living with dementia in Ontario are unavailable, the available statistics call into question the extent to which the under- or unavailability of PSWs contributes to these losses, and whether mitigating gaps in PSW availability would bolster not only future employment productivity among caregivers, but other domains of financial risk, including caregivers’ overall income levels and personal health.

In Ontario, publicly funded home care is currently organized and provided by Home and Community Care Supports Services (“HCCSS”), which is a group of 14 decentralized units, or organizations, that are held accountable by Ontario Health for delivering home and community-based programs and service (Connecting Care Act, 2019). Prior to April 1, 2021, HCCSS were known as Local Health Integration Networks (“LHINs”) which had a broader remit than HCCSS including home and community care, as well as long-term care and hospital care. However, in both the LHIN and HCCSS contexts, the process of acquiring home care is the same. Briefly, individuals requiring home care—irrespective of having previously had a hospital visit—and/or their caregivers can self-refer or be referred by their primary physician to their local HCCSS. A care coordinator (case manager) is then assigned, who works closely with primary care physicians, care recipients and their caregivers to determine what is needed and arrange necessary services (Government of Ontario, 2022b). Homebased services provided by home care workers (e.g., PSWs, nurses or social workers) for which home care recipients have been deemed eligible are covered under the Ontario Health Insurance Plan (OHIP). The extent of service (e.g., number of hours of personal support work, or number of visits by a physical therapist) is determined following an assessment of need conducted by the care coordinator. Home care recipients (or, by proxy, their caregivers) are responsible for paying for any additional services that exceed the service maximum set by HCCSS.

Informed by welfare state theory and notions of financial risk, the objective of this qualitative study is to use Ontario as a case-study to explore how unpaid caregivers of persons living with dementia receiving home care experience financial risk in navigating publicly funded home care provision. Understanding how financial risk is experienced across various dimensions of navigation will better inform public approaches that aim to mitigate these risks.

## Methods

### Overview of qualitative theory

This study is grounded in critical realism, which assumes that our understanding of a phenomena is shaped by the complex interaction between individual values, beliefs, experiences and knowledge, structural factors, including social and cultural normal and physical environment (Connelly, 2001). Critical realism is a common knowledge paradigm in health and social policy research because of the underlying assumptions that individuals’ interpretations of a phenomenon (e.g., a policy) have influence over the nature of social change (Gilson, 2012). Accordingly, the assumptions that underpin critical realism are useful in understanding policies across welfare states. Specifically, underpinning welfare states is a set of rules, morals and ethics, and modes of thought concerning the distribution of social benefits to members of society. However, the way in which individuals interact with constituent policies differs. Together, these interactions cohere and create a constitutive meaning for policies of focus (Bhatia & Orsini, 2014). Critical realism therefore facilitates an analysis of welfare states that does not sacrifice the unique nature of individuals’ lived experiences interacting with policies within different types of welfare states. This, in turn, enables the application of interpretive methods and analytic approaches that aim to unearth these lived experiences (Connelly, 2001).

Drawing upon this paradigm, this study is informed by interpretive description inspired by Schützian social phenomenology. Schütz’s approach to phenomenology is descriptive as it seeks objective knowledge through accurate descriptions of subjective experience (Shaw & Connelly, 2012). This compliments critical realism, which accepts that the welfare state, though representing one structural reality, can be experienced differently by individuals. Broadly speaking, this approach also aligns with studies concerning caregiving. Schütz posits that social interaction plays a key role in establishing meaning and knowledge for people; people learn what phenomena (e.g., caregiving) mean through memories of past experiences and how others project their own meanings in the world. We know that caregiving is a phenomenon that does not occur in isolation; caregivers are linked by their many “positions” in the world (including positions determined by gender, age, occupation, socioeconomic status, language, country of origin), and their social interactions with each other (McGregor and Ronald, 2011). These ideas frame the approach taken in this study concerning qualitative data generation and analysis.

### Recruitment

This study was approved by the Research Ethics Board at a large Canadian university (#20126). Participants were recruited from a population-based, anonymous cross-sectional survey conducted by our study team between August and December, 2020. This survey explored the financial risks of unpaid caregiving during the COVID-19 pandemic. Detailed approaches to developing this survey have been described elsewhere (Marani, Allin, & Marchildon, 2021). At the end of the survey, caregivers of persons living with dementia receiving home care were asked if they would be interested in being contacted to participate in a one-hour follow-up phone or video interview to gain a deeper understanding about how they experience the costs of caregiving and how these experiences can be understood given the functions of financial risk protection policy in Ontario. Caregivers were eligible to participate if they were: 1) a resident of Ontario; 2) 16 years of age or older (Ontario’s legal working age); 3) providing care on a voluntary basis to someone living with Alzheimer’s disease or related dementia at home (i.e. not living in a residential long-term care facility); and 4) proficient in basic conversational English or had access to a translator in the household. Based on the information provided on the survey about this interview, interested participants provided their informed consent to be contacted by the principal investigator by entering their email address. Those who provided an email address were then sent a consent form to review outlining study details. All caregivers who agreed to participate were interviewed and provided oral consent before participating.

### Data collection

Interviews were conducted between August and December 2020 based on an interview guide informed by study goals (see Supplementary Table 1). Questions were semi-structured in nature to enable the interviewer to engage in emergent discourse with each participant, and to allow insights from earlier interviews to guide and improve subsequent interviews. The interview guide was co-developed by the principal investigator and three volunteer caregivers outside the study team who were not involved as study participants. The interview guide was also validated by two qualitative methodologists at the institution where this study took place. Based on feedback, this interview guide deliberately used lay language to facilitate the participation of those who knew English but lacked a strong mastery of the language. This was important as ensuring both the researcher and informant understand the phenomena in question would prevent erroneous interpretations of meaning in analysis. All interviews were conducted over the phone or video conference (Zoom) given the constraints of the ongoing COVID-19 pandemic.

### Data analysis

All interviews were audio recorded and transcribed by the principal investigator. All identifying details were anonymized. Transcripts were then analyzed following analytic approaches in phenomenology to understand the nature of the experience of caregiving. Three transcripts were manually coded inductively by the principal investigator then validated by co-investigators through a consultative process of coding techniques and a discussion of example codes. In line with phenomenology, this manual coding process involved descriptive, segmental open coding based on the nature of participants’ experiences (Sandelowski, 1995).

A codebook (see Supplementary Table 2) was then generated deductively by re-organizing codes derived from three transcripts into broader categories (Sandelowski, 1995). These categories, which reflect how caregivers experience the financial risks of caregiving, were informed by a framework adapted by the study team that organizes financial risk across three broad domains, including risks to overall income, employment productivity, and personal health. Subsequent transcripts were then coded using NVivo 12 based on this codebook. Underpinning phenomenology is the importance of a coding system to identify commonalities between interview participants’ experiences; however, phenomenology also recognizes how the depth of experiences may be lost in a codebook generated from the coding of a small dataset (i.e. only three transcripts) (Larkin et al., 2006). Hence, in the analysis of subsequent transcripts, the codebook was left open to the possibility of new codes that reflected the nature of the experiences of caregiving in relation to financial risk.

### Rigour and validity

To ensure rigour and validity, this study incorporated: 1) engagement in reflexive dialogue with members of the study team about assumptions and emerging descriptions of the research data (including being open and transparent about the principal investigator’s own position as a caregiver-researcher); 2) participant follow-up to expand and clarify the meanings of descriptions as they relate to participants’ experiences; and 3) a discussion between co-investigators to address any lack of correspondence between excerpts from transcripts (quotes) and their respective codes.

## Results

In the corresponding survey from which interview participants were derived, 65 caregivers provided care for someone living at home with dementia. Of this group, 33 caregivers agreed to be contacted about participating in a one-on-one follow-up interview, of whom 24 agreed to participate in an interview. Though we did not strive for theoretical saturation as this is not normally an aim in interpretative description informed by phenomenology (Saunders et al., 2018; van Manen et al., 2016), the number of participants derived from our brief recruitment period is considered sufficient for the purposes of rigor and validity in qualitative reporting (Gentles et al., 2015; Cohen et al., 2000).

For context, Table 1 presents a demographic profile of participating caregivers, including their age (range 24–81), gender (*n* = 20 women, *n* = 4 men), and employment status (evenly split between working and not working). This table also summarizes the number hours of unpaid care provided per week (range 7–168), as well as whether or not unpaid caregivers were receiving care support from a PSW; eight do not receive help from a PSW. Participating caregivers were providing care for either a spouse (*n* = 9), parent (*n* = 12), friend (*n* = 2) or grandparent (*n* = 1). With the exception of three caregivers, all participants were white (Caucasian).

**Table 1.** Interview participant details.

Participant ID	Length of interview (mins)	Age	Gender	Employment status	Hours of care provided/week	Receipt of PSW	Care recipient
001	90	77	Woman	Retired	40	Yes	Spouse (Husband)
002	84	54	Woman	Self-employed	7	No	Parent (Father)
003	55	48	Woman	Self-employed	20	Yes	Parent (Mother)
004	47	56	Woman	Employed	20	Yes	Parent (Mother)
005	65	72	Man	Retired	168	No	Spouse (Wife)
006	47	77	Man	Retired	84	Yes	Spouse (Wife)
007	94	24	Woman	Employed	70	Yes	Parent (Father)
008	46	63	Woman	Retired	154	Yes	Spouse (Husband)
009	59	74	Woman	Retired	168	No	Friend (Woman)
010	82	44	Woman	Self-employed	38	Yes	Parent (Mother)
011	51	73	Woman	Retired	56	No	Spouse (Husband)
012	82	76	Woman	Retired	40	Yes	Spouse (Husband)
013	43	58	Woman	Retired	24	Yes	Spouse (Husband)
014	71	43	Woman	Self-employed	90	Yes	Parent (Mother)
015	60	69	Woman	Retired	7	No	Spouse (Husband)
016	62	66	Woman	Retired	10	Yes	Parent (Mother)
017	70	61	Woman	Employed	28	Yes	Parent (Mother)
018	92	42	Woman	Long-term disability	70	No	Parent (Mother)
019	64	40	Woman	Self-employed	20	No	Grandmother
020	46	66	Woman	Retired	5	Yes	Parent (Mother)
021	71	36	Man	Unemployed	35	Yes	Friend (Woman)
022	31	81	Man	Retired	168	Yes	Spouse (Wife)
023	62	61	Woman	Retired	168	No	Parent (Father)
024	66	56	Woman	Employed	168	Yes	Parent (Mother)

The average length of interviews was 64 minutes, and all participants were compensated for their time with a monetary (cash) honorarium of CAD $30 sent via e-transfer. This is just above the mean ($28.50/hour), median ($25/hour) and mode ($25/hour) honoraria for interview participation compensated with cash in Toronto, Ontario (Cheff, 2018).

Caregivers noted experiencing financial risk across three broad dimensions associated with navigating publicly funded home care: 1) in receiving information about home care availability needed in the future, specifically supplemental care from a PSW; 2) in requesting or receiving support from a PSW, particularly flexibility around hours and extent of support; and 3) in maintaining consistent access to quality support. Select quotes are presented in the results below and a glossary of additional salient quotes is presented in Table 2. All caregivers reported their experience navigating home care provided by their “LHIN”, not “HCCSS”.

**Table 2.** Glossary of salient quotes across Themes.

Theme	Participant ID per results	Quote
Learning about publicly funded home care	INT015	I need to know what’s available in terms of help, how I get access to it, and how it gets paid for.
	INT018	At [local hospital], there is a geriatrics inpatient three-hour assessment that cover [s] everything to do with the individual’s life. And that was a game changer for my mom, my dad and I went with my mom […] I think that needs to be done exactly when people turn 65, if not sooner. They talk about […] seeing a financial advisor, […] having your will prepared, talking to your kids about this […] you almost need a third-party person getting involved, because nobody else can do for you.
	INT014	I Find that doctors are often [focused on] two-three symptoms because of the time they have. And therefore [they are] not looking at the big picture. […] I experienced this with my father […] in terms of his medical issues. […] They’re looking at giving a band aid for this and giving a band aid for that, but not looking at the bigger picture of it all. And I feel like most of the time [in the office] should then be spent […] connecting with different agencies and organizations to [figure out] what are we dealing with […] and how [to] best manage and care for this. Because […] when you ask me questions about, you know, am I tapping into any of these resources, there may be resources that […] are accessible to me […] however, I’m not aware of them. And I think that that’s sort of the biggest piece [for] the medical community: What is it that we’re dealing with that how do we respond to that.
	INT018	There’s not enough time [in] a day and all this stuff takes so much time. […] I navigate the system as best I possibly can. I Have the English language to protect me, but I’ve been in hospital waiting rooms where I feel bad for other patients [for whom] English is not their first language. They have no clue what’s going on in this world. No clue. […] I feel bad for people I really do. But I feel like there’s not enough social workers in the planet to be able to help out with how to navigate. There’s lots of information out there. I just don’t think it’s being presented. It’s almost too late when it’s being presented to you.
	INT014	I think that in a perfect—in a better world—offering those pieces would be helpful. However, if it’s going to take away from achieving their primary goal [medical care], I’d rather them achieve the primary goal first.
	INT018	There’s almost too many of them [supports and services]. Nobody has a clue as to what there is available. It makes no sense. And there’s no marketing […] like there’s no marketing budget to be able to let us know. Even my family doctor said there’s so many different ones [supports and services]. […] you just got to call them and find out what’s the right fit for you. And I’m like, “why does there have to be eight different services that do the same thing”
	INT017	When the OT [occupational therapist] came and said I needed a hoyer lift […] she [the OT] said, “your mother might qualify through March of dimes”. I’m like, “oh, you’re kidding.” so she goes, “yeah”, but […] I needed to get her [my] income statement. So I gave her the [income statement and] she [said I was] $2,000 over. I’m like, “are you kidding?” she [my mother] makes nothing. She was a pensioner. She makes nothing [she] still gets nothing. I don’t know how much less she could make. And that was it. [So] I said, “well, are there any other people that might cover it?” she didn’t know. So now I’m on my own. I Gotta […] see if somebody else will cover it. It really shouldn’t be the responsibility of the family. There’s no closing up the loop. They just go “well, this is the one I know and I don’t know any other.”
	INT013	The government is providing the support through LHIN, right? So LHIN knows about these programs, but they don’t inform the people about it. I mean, I came to know through that nurse, otherwise, I wouldn’t know about this program. So at least give that option to the patients—[that] there is a program like this where you can avail [yourself]. Give the information. I’m not saying that everybody [would be] eligible for it, but at least give the information to say that [it] is available.
Requesting and receiving support from a PSW	INT014	The hours that I work would be the hours that she was sleeping. And so, in order for me to be up and able to work at the times that I could work meant, one, I wasn’t there to help her get ready to go to bed. And two, I wasn’t able to get up at the hour that I was required to get up in order to go to work. So that was in 2018 when that started. [After,] I started to gradually cut back in [work] hours. And that progression has continued as her disease has progressed.
	INT020	I don’t know what they [personal support workers] are doing with their time. They’re not allowed to prepare food. They’re not allowed to […] give her her pills […] they have kind of hand them to her and watch her take them. But they’re not even doing that. I Went in last week, and there was a note sitting there on the on the table, saying, “take your pills.” Take your pills, and then they left. So I mean, literally, they’re in my mom’s apartment for probably less than 2 minutes of a 30 minute entitlement.
	INT003	About six months in I had to push quite hard […] to not have a male [personal support worker]. I said that that’s completely inappropriate. I don’t need to get into the why. But all you need to know is there’s a reason and there’s a history and that’s it. So no male. So I finally got a supervisor [case manager or care coordinator] that said, “understood". And from there on, I didn’t have an issue, but a few of the supervisors—my sense was they were just young—didn’t really get what I was talking about and so, that lack of experience […] they really couldn’t empathize what I was trying to say.
	INT023	I don’t want to shower my dad. It’s a personal thing for me and it’s private for him. So we’re gonna have a PSW [personal support worker] come in to give me a couple hours at that time where I can have some time to myself […] go out without worry, like, [to] get groceries. But I’m always nervous when I leave him home alone.
	INT010	[It] took me a while to find a Korean [home care worker], which it took a shout out on facebook to my friends […] because once again, nobody I know has to deal with this. And then, luckily, one of my friends is a social worker that was working at [a large local hospital]. And she saw something in the newsletter about a Korean caretaker. […] so from her, by chance, I was able to find a lady that would just come and hang out with my mom for 3 hours, twice a week. They would watch some Korean TV. And [sing generational songs] since she’s just a little bit younger than my mom, maybe like five to seven years younger than my mom. And like that caretaking was […] $26 an hour. So I would do that 3 hours, twice a week. […] It also gave me some time to spend at work because […] I work really long hours, and then I would maybe have dinners with friends and stuff. So my life was mostly work. So for me to try to get back quickly for my mom left me not too much time.
	INT024	I used to have to travel back and forth to give my mom showers and things because they [the LHIN] only gave us 1 hour [of personal support work]. And it took her [the care coordinator] till last year, like four or five years later, to give us more help, more showers, and things like that. So an hour for five days a week. I told them that [was insufficient] at the beginning. And they said, “oh, well, you know, when you go back to work, give us a call. And we’ll see if we can help.” […] They’re depending on me.
	INT020	I don’t want to do this, you know? I Feel like I’m being selfish, but I don’t want to do this. I do it because she’s my mom. And I have to at this point, but I don’t like feeling that I have to, you know?
	INT017	I’m not afraid to step on toes or give them the indication I’m not happy. […] recently we got a hoyer lift [because] the problem we’re having is the PSWs [personal support workers] coming in [and saying,] “I can’t do this, I can’t do that. I’m not allowed to lift…” […] so you’re always getting push back. So then when you talk to the LHIN, they send the OT [occupational therapist] and the OT comes in and says, “well, you know, you really need to get this [a hoyer lift].” so you go “well, how much is that? … oh, that’s 3500? Okay, so if I get that we’re good? Okay,” so then you run out and you get whatever it is they tell you to get. Then the PSW comes in and says, “no I can’t do it, because—” I’m telling you, it’s just so challenging and not very flexible to families. And like I said, it’s only because of the type of person I am that I do what I do. I’m sure there’s thousands of families out there who just go, “well, we can’t afford it.” so now their loved one is in bed all day because they can’t afford the wheelchair or they can’t afford that, this or that or the other. And then the PSW that comes in just rolls them over and washes them in bed and dresses them and out the door they go. They know when they come to the home, that they’re there for an hour. You would be surprised how many times I don’t a) get the hour, and b) they refuse to do certain things. And [now] I don’t even have them come to be perfectly honest. If I’m going in to be there for the day with my mom. I just tell them [the LHIN] to like cancel the service for the day.
	INT024	I applied for [a bed] and they said we didn’t qualify because my parents pay for internet and TV […] things like that. [So] I said, “well, I don’t know what we’re gonna do, because they [my parents] don’t have any money in the bank [and] I don’t have money. My mum is in dire need of a bed because she can’t get up and down the stairs anymore —the bedrooms are upstairs.” so he [the care coordinator] heed and hawed about it, then came back to me and he said, “you know, I can actually…I shouldn’t do this, or I don’t normally do this, but I’m going to approve it.”
Maintaining access to PSW care	INT003	The caregiving agency we were with had, as I understand it, a bit of a different model. They tried to keep the caregivers [home care workers] the same, which was really helpful. Because mom really did not like when, of course, when there were different people caring for her.
	INT017	I’ve had caregivers [home care workers] come in that that [have] had ideas […] and [I] would think, “are you out of your mind,” but it works for my mom. So as long as it works for my mom, I stay out of the picture. Because I know when my mum’s not liking somebody [is] when the aggression comes out—when she’s sitting, she will try to raise her leg to kick, or she’ll wave her hand, [to imply] “get away!” Or she’ll tell them, “stop, stop!” and then she shuts down. So, she senses certain things and I pick up on that and I know whether or not [she’s happy with] the care that she’s getting. [Since] she can’t really articulate […] I just say, “listen, this is probably not working out, because that’s why she’s behaving the way she’s behaving.”
	INT023	I wouldn’t want to push him [my dad] any longer with a stranger. And the thing with not going with the LHIN and maybe going for a private service is we would more than likely get the same person each time from a private service. Like with my mother [never had] the same person [bathe her], and that was kind of hard for my mom, but I couldn’t do it, so…
	INT013	Because of his condition and COVID, I’m spending more on his things. Like physiotherapy is out of my pocket. It is $400 every week […]. Then for his incontinent supplies [it’s] around $50 every week—diapers and all. And if I had a PSW [personal support worker] that would be another expense, so that’s why I stopped our PSW [and] now I’m taking care of him.
	INT007	It [COVID-10 pandemic] has affected [us] a lot. […] We’re having PSWs [personal support workers] come into my home every single day, and as well as nurses. [This] poses a significant health concern because those PSWs are going into other people’s homes, into long-term care homes, and [then] coming into my home. So it definitely is something that I keep at the back of my mind. […] I don’t know how many come into my home on any given week, but just different people. And they don’t take precautions of doing any pre-screening questions before they enter, so they just come in just naturally on their own. And I’m afraid about if a PSW were to have it, and contracted it, how serious and how detrimental that would be to my dad, because if he were to get COVID, it would be game over.

Dimension 1 Information about the availability of publicly funded home care supports cannot anticipate future care needs along the dementia care trajectory, compromising caregivers’ ability to be financially prepared.

Caregivers were highly perceptive that their care recipient’s dementia diagnosis would be characterized by immediate and future expenses as the disease progressed. They were also aware of how these changing needs would shape acute and ongoing costs of caring, and the importance of being aware of care supports available, particularly access to PSWs, during these transitions.

*So yes, we can afford his dental work and we paid for it. And that’s not something I’m expecting to have to pay again anytime soon. So you know, the expenses kind of go up, and then they […] level off for quite a while until something else happens. [INT011]*

Accordingly, caregivers described the immense value of PSWs in supporting care recipients’ activities of daily living (e.g., bathing, lifting and feeding) that caregivers were otherwise unable to do on their own particularly during complicated transitions which involved demanding care tasks. Caregivers reported that they first sought support from PSWs through their LHIN.

For some caregivers, awareness that publicly funded home care provided by a PSW exists came from previous or current experiences caring for another family member. For others, however, realizing a PSW would be needed to support increasingly challenging care activities arose when faced with that dilemma, by which time finding a publicly funded hours of personal support work on short-notice was challenging, and paying for a PSW privately was otherwise cost-prohibitive.

*The only time I […] knew about anything was because my mother was hospitalized. And then of course, they hook you up with a social worker. And that’s how you get to find out what’s available. If you don’t, then you have no clue. And if you don’t go online and do your research, again, you have no clue. So anybody that I’ve run into that is having challenges [with] care. You know, […] I’m one of the first ones to say, listen, call the LHIN, you got to get a caseworker [care coordinator]. And you have to get somebody at the LHIN and get your […] parents taken care because there are services. [INT017]*

Caregivers attributed these struggles to a home care system that is poorly integrated (streamlined) and unresponsive to current needs. Caregivers suggested the need for a designated individual (a “middle-person”) to offer financial advice specifically [INT015], and bridge informational gaps between caregiver, care recipient, and publicly funded home care available [INT018], irrespective of whether or not these supports would be needed later on [INT014]. This was echoed by other caregivers who highlighted the immense value of a designated point person particularly for caregivers who do not have a computer, or, for cultural or language proficiency reasons, would not ask for help [INT018]. However, others suggested that, although having ongoing financial advice was useful, it should not detract from medical care [INT014].

*[…] it is the government […] providing the support through LHIN, right. So [the] LHIN knows about these programs, but they don’t inform the people about it. I mean, I came to know through that nurse, otherwise, I wouldn’t know about this program, so at least give that option to the patients to say, you know, what, there is a program like this […] Give the information. I’m not saying that, you know, everybody is eligible for it, but at least give the information to say that […] this is available … [INT013]*

*Yeah, I mean, if I’m calling for help, I need it now. If I’m desperate enough to phone a helpline, I need it now. I don’t need it 24 hours from now. [INT008]*

Drawing on these experiences, caregivers conveyed that the existing system of care coordination (case management) does not recognize the importance of having conversations about future care and caregiving needs, including financial planning and major caregiving expenditures such as home care provided by a PSW. Caregivers noted their one LHIN-assigned care coordinator (case manager) would offer advice on what they needed (Hoyer lift, bed, etc.), but was often unable to answer important follow-up questions, for example where to go among the myriad of, often redundant, services available [INT018], the most cost-effective options, and how to pay [INT017]. Care coordinators were also unable to answer questions concerning future care, for example, around eligibility for supports and services [INT013]. This hindered how proactive caregivers could be because the information received was often based on the care recipient’s current state.

*[…] when my husband fell out of bed and he was lying on the floor, I phone [d] 911, I didn’t know what else to do. And I was told by the LHIN, “well, you can’t continue to do that [as] that’s not a good use of resources. You’ve got to have a neighbor that you can [call] on.” [But,] my husband weighed 250. Like it’s gonna take more than one neighbor. And at three o’clock in the morning, that’s probably not [possible]. So it was like, yeah, we’re here to help you to a certain extent. [INT008]*

Some caregivers felt strongly about appropriate case management to manage the financial risks of caregiving.

*[…] I would love to be able to have a social worker assigned whenever […] Sometimes you need a middleman [but] I don’t even know where to go for that. I don’t want to be [in] that […] role [for] my parents life, right? […] I’m the bad person [when] I’m the one who’s, you know, making [their] decisions. It’s not fair to me. [INT018]*

As evidenced by the above quote, these caregivers did not want to have to manage the mental math associated with navigating future care expenses and available income to pay for these expenses. Accordingly, they spent considerable time researching options, which they likened to an opportunity cost.

Dimension 2 Lack of flexibility around hours and extent of support provided by a PSW imposed greater responsibilities on caregivers, in some cases compromising their employment productivity.

In Ontario, caregivers described the process of acquiring a PSW from their LHIN as challenging. Assessments conducted by a LHIN care coordinator to determine need for a PSW often yielded fewer hours of subsidized personal support work than caregivers requested. Other caregivers were denied hours at a specific time of day (e.g., nighttime), and in some cases, were denied any personal support work at all on the basis that the care recipient did not need help.

Caregivers felt this needs assessment approach was inconsiderate of the unique context of caregiving for someone living with dementia. This assessment was often conducted on a “good day”, undermining the hidden realities of caregiving for someone living with dementia and the constant care and monitoring (daytime and nighttime) involved.

*We definitely wanted the LHIN assessment just to see and make sure that […] she was safe to be alone. That was kind of more of our concern […] did she need to move into [our house] now? Because, as family, it’s hard [for us], right? We see a drastic decline, but from an outside perspective, it’s not as serious maybe as we might feel it is […] and I also think when [the LHIN] came, she had [been having] a good day. […] so there’s good and bad days and the bad days are quite different than the good days. [INT019]*

Further, although LHINs (now HCCSS) perform re-assessments annually or when there is a significant change in condition, caregivers noted that outcomes of assessments (e.g., number of hours of personal support work allocated) were based on current needs only, and not anticipatory of the rapid transition of needs characteristic in some types of dementia. Caregivers described the need for flexible and adaptable hours to account for abrupt changes in dementia symptoms, including shifts in sleeping patterns (sleeping during day and awake at night), the sudden onset of wandering, and needing assistance with toileting and bathing that they could otherwise assist with on their own at first, but then could not as the dementia progressed. Without this adaptability, caregivers experienced disruptions in productivity at their places of employment [INT014] and concerns that they would have to pay for this care support out-of-pocket later on.

*The expectation is her needs will be greater, right? As time goes on, more 24/7 [needs], waking up possibly more, we don’t know. But there’s no more […] help available without me [having to] to pay for it. So it’ll be all down to us to do that. [INT016]*

Relatedly, caregivers reported that the number of daily or weekly hours of subsidized personal support work received was insufficient. This, coupled with restrictions by the LHIN on care activities that PSWs could perform beyond bathing, room tidying and dressing (e.g., meal preparation) [INT020], and, in fewer cases, the unavailability of PSWs of a specific gender [INT003, INT023] or cultural background [INT010], meant caregivers had to seek and pay for additional hours of personal support work out of their own pocket, irrespective of whether or not they could afford it. Other caregivers purchased supplemental private insurance to fill these deficits.

*But I’m telling you, […] when you have to make these decisions about what they need—like, I needed to get a hospital bed— […] you can’t not get it. So it’s almost like, you know, I’ll go work on the street if I have to [in order] to be able to afford this. [INT017]*

Retired and non-working caregivers living with their care recipient reported receiving less hours from the LHIN under the pretense that they would be present at home some [INT024] or most of the time. These caregivers found it frustrating that the LHIN was leveraging their unpaid labour as a rationale for providing less subsidized home care by a PSW.

*Well they [the LHIN] has not given me any support. […] In fact, they have used the fact that I’m living here as an excuse not to bring more workers in. Essentially, that was part of the reason that they wouldn’t budge from seven visits a week. So, it took a bit of arm twisting and reassessment of [care recipients’] … after she had to go to hospital from a trip [fall]. Only after that, could we get 14 visits in a week. They considered […] me just like a resident, essentially, which, I guess blurred the lines. They felt that she would be more at risk if she were living by herself… [INT021]*

In light of these issues, caregivers often paid for additional hours of personal support work out of their own pocket out of necessity (e.g., because they had to work during the day), or filled these deficits with their own unpaid labour despite the perceived physical and emotional burden this imposed. Despite these challenges, caregivers noted no aversion to filling these deficits themselves, particularly if the care recipient was their parent or spouse [INT020].

Others felt the formal medical system, including palliative care, offered a more standardized pathway for receipt of a PSW or direct admission into a residential long-term care facility. Some caregivers expressed a desire for their care recipient to end up in hospital—a universal health service provided free-of-charge to all based on provincial residency—as this would automatically put their care recipient on a waitlist for residential long-term care, or initiate a process for receiving additional hours of home care by a PSW.

*I hate to say this, we honestly hope that she ends up back in the hospital, and [then] we can refuse to take her home because she can’t care for herself. So, therefore, she would just stay in the hospital until there was a bed at a nursing home for her. That’s where we are at this point. And that might sound cruel and unusual, but it’s, you know, it’s almost like wishful thinking. [INT020]*

As a result of these challenges receiving support from a PSW, caregivers noted becoming advocates for their own care recipients to fulfill their needs.

*I don’t mind ruffling feathers […] I basically wade through the system, like a bulldozer, at first being polite, considerate and respectful. The second thing is being resourceful in terms of negotiating compromise. And the third one is going ballistic. [INT012]*

As evidenced by the above quote, caregivers who self-identified as being strong-willed or persuasive felt they could obtain what they needed (e.g., additional hours of personal support work or a bed/lift) by negotiating or arguing with their care coordinator [INT017, INT024]. In a few cases, this advocacy stemmed from prior experiences caregiving and navigating the LHIN.

Dimension 3 Challenges maintaining consistent access to quality publicly funded personal support work necessitated caregivers to hire a PSW privately, or re-arrange their working hours to fill support gaps.

Some caregivers noted that their care recipient did not want any care from a PSW. Caregivers noted this resistance was rooted in a number of factors. One factor was their care recipient’s stubbornness, which, when exacerbated by dementia, made them hostile and aggressive toward PSWs. A second factor was the perception that the care recipient was still capable of performing activities of daily living independently. Additional factors contributing to the reluctance to accept external support from a PSW included a desire to remain in control of their livelihood, general denialism of their dementia diagnosis, and a hyper-awareness of the cost of their own illness. For example, one caregiver’s father felt petrified of having a PSW, recognizing the cost of that care and not wanting his daughter to bear that financial responsibility.

*[My parents’ expenses have] all been out of my own pocket. […] like, the roof […] was leaking in the garage and leaking all over the bathroom. My parents [said], “Oh, no, we don’t want to spend the money.” My dad is in the mindset of […] he’s petrified […] that my mum’s gonna need extra services because of her dementia, and he’s […] petrified of running out of money. […] He’s not the only one. I’ve had this conversation with many of my friends, parents, and old coworkers. […] It’s such a common thing. We just don’t talk about these things. [INT018]*

Caregivers also spoke of preferences in having continuity with their PSW [INT003] and concerns with having different PSWs supporting their care recipient at any given time. The involvement of inconsistent PSWs was the result of PSWs requesting reassignment, resulting in a service provider organization—which hires and contracts out PSWs on behalf of LHINs—sending a different PSW to a care recipient’s home. This lack of care continuity exacerbated dementia-related confusion for care recipients. In some cases, this confusion resulted in acts of aggression and violence towards PSWs they did not recognize, particularly by those at later stages of their dementia diagnosis [INT017]. Given the unpredictability of dementia, caregivers found themselves having to spend considerable time updating PSWs of their care recipient’s behavioural changes and emerging needs. For care recipients with inconsistent PSWs, caregivers felt exhausted having to repeat this information to multiple PSWs. Consequently, in the interest of consistency in care, caregivers opted to hire a private PSW, paid for fully out of their own pocket [INT023], who they found on social media or through a friend. Other caregivers opted to scale back work hours to care full-time.

Furthermore, several caregivers were dissatisfied with the quality of personal support work received. This dissatisfaction was attributed to: PSWs doing less work than the time they were being paid for, leaving early, and PSWs not being trained to care for persons living with dementia, for example, by providing emotional and social support (chatting). This meant caregivers found themselves having to be present at all times to monitor their PSW who was otherwise there to provide respite for the caregiver or enable them to continue working at their place of employment.

*[The PSW] would come for 1 hour, but they [would] do just half an hour work. So they would just come home [and] give him […] just a bed bath […], change him […] and go out. That’s it. Nothing else. So, you know, they’re not providing any cognitive help or anything to him. So, I think we need somebody who knows dementia patients to provide [for] them, you know? At least to do half an hour of cognitive exercises or something of that sort. [INT013]*

Caregivers also referred to the unique circumstances associated with providing care and receiving services during the pandemic. Specifically, some caregivers were reluctant in accepting publicly funded home care provided by a PSW during the COVID-19 pandemic, citing concerns with transmissibility between homes PSWs worked at. Accordingly, several caregivers who previously paid for personal support work (e.g., additional hours beyond those publicly subsidized or hired privately) cut back on PSW hours or stopped accepting PSW help from their LHIN, which, for some, resulted in considerable cost-savings. These savings, however, were replaced by other COVID-related care expenses [INT013] borne of out of pocket by the caregiver. Some caregivers accepted this risk, citing the ongoing need for this care [INT007].

## Discussion

In this study, we found that caregivers experience financial risk stemming from navigating publicly funded home care across three dimensions: 1) receiving anticipatory information about publicly funded home care available, specifically PSWs; 2) receiving flexible hours of support from a PSW; and 3) maintaining consistent access to quality support from a PSW. Broadly, there is a clear need for publicly funded home care provided by a PSW to help caregivers with complicated and unique care activities associated with caring for someone living with dementia. However, challenges acquiring sufficient hours of personal support work or maintaining existing support compromised working caregivers’ ability to stay gainfully employed, and, accordingly, limited their income-generating potential. Other financial risks among caregivers emerged from having to hire a PSW privately to fill deficits in publicly funded personal support work, and/or to ensure consistency in care. These hours were paid for at the caregivers’ expense, which was cost-prohibitive and unsustainable.

Taylor and Quesnel-Vallée, (2017) describe the complexities unpaid caregivers in both Canada and the United States experience in navigating health and social care as unique structural burdens. They specifically point to time-related challenges associated with care planning as rooted in the discontinuous and fragmented nature of available services including care coordinators and PSWs, which is consistent with our findings. Our findings are also consistent with emerging international research, which has shown that a lack of formal care availability has contributed to psychosocial and financial hardships for unpaid caregivers of persons living with dementia, including difficulty maintaining employment during caregiving (Lindeza et al., 2020).

Results from this study go one step further by describing how perceived shortfalls in the home care sector experienced by homebased caregivers of persons living with dementia manifest as financial risk. We suggest that the structural burden associated with a system that does not consider the unique nature of dementia in allocating publicly funded hours of personal support work has translated into financial risks, including caregivers mobilizing limited personal funds to hire a private PSW to fill public deficits, or forgoing assistance from a PSW altogether. These observations are similar to the concerns raised by parents of children with medical complexity; the significant demand for care, often required 24/7, and challenges navigating publicly funded services that do not meet care needs necessitate expenditures incurred out-of-pocket by caregivers to fill these gaps (Garnett and Browne, 2016; Genereaux et al., 2015).

This study’s findings also provide a more up-to-date understanding of financial risk in the context of the COVID-19 pandemic. Evidence from the local context is scant concerning how single-site employment restriction policies (restrictions imposed by PSWs’ employers on PSWs working in multiple settings) has impacted unpaid caregivers’ financial well-being. We found that, in fact, caregivers opting out of home care support during the pandemic or receiving less support due to restrictions on PSWs entering homes has saved some caregivers money even though the COVID-19 pandemic has affected caregivers negatively in other ways (e.g., their mental health) (OCO, 2021).

These findings have important implications concerning policy and practice. The ability of some caregivers to protect themselves from shortfalls in publicly funded personal support work, for example, paying for a PSW hired privately or self-advocating for additional PSW assistance from HCCSS, may perpetuate inequities in access to home care, driving other caregivers toward greater financial risk. This is particularly important in an already resource-constrained environment exacerbated by the COVID-19 pandemic, which has resulted in a mass exodus among PSWs working in the home care sector due to: workplace hazards and low pay (Marani, McKay, & Marchildon, 2021), struggles by service provider organizations in retaining and recruiting PSWs (Casey, 2021), single-site employment restriction policies (Zagrodney et al., 2021), and school closures during the pandemic requiring PSWs to take on childcare responsibilities (Hapsari et al., 2022; Zagrodney et al., 2021).

Useful policy lessons can also be gleaned from these findings. Questions concerning the sustainability of unpaid caregiving to address shortfalls in health human resources have prompted discussions about how to develop approaches that mitigate financial risks of unpaid caregiving. These include public cash transfers for caregivers (Flood et al., 2021), and directly funded (DF) care, which provides caregivers with a budget to arrange their own home care in lieu of receiving publicly arranged services (Kelly et al., 2021). In the context of PSW deficits, these models would allow caregivers to pay for additional care support if needed, thereby reducing inequities associated with certain groups being able to pay for a private PSW. In some cases, these models may also function as monetary compensation for time taken off work for caregivers who, given the nature of dementia care, may opt not to hire a PSW.

### Limitations

Caregivers in Ontario are a highly heterogenous group representing a variety of ages, cultural backgrounds, and experiences. While the goal of qualitative research is not to claim that any specific empirical finding from a sample of participants can be transferred or generalized to an entire population (Polit and Beck, 2010), we do acknowledge that a more diverse sample of participants would offer even further benefit to the applicability of the findings across caregiving circumstances, and therefore confirmability among other caregiver groups.

## Conclusion

Unpaid caregivers of persons living with dementia experience several challenges navigating publicly funded home care, including challenges receiving information about supplemental care support from a PSW; receiving flexible hours of support from a PSW; and maintaining access to quality support from a consistent PSW. These challenges mean caregivers may have to pay for additional hours of personal support work out-of-pocket, which may be cost-prohibitive. Against the backdrop of COVID-19, unless caregivers are properly supported, it may be unsustainable to continue to rely on their unpaid labour to address shortfalls in the availability of PSWs, particularly in the context of dementia care. Addressing the conditions that shape the organization of publicly funded home care that are contributing to financial risk among caregivers is therefore an important policy issue. Findings from this study are useful in ongoing home care reform efforts to ensure caregivers are adequately protected from financial risk. Indeed, lessons from this study can also be applied in other jurisdictions with Canada, and in other countries where there is a growing dependence on unpaid caregivers in the context of a home care sector struggling to recruit and retain PSWs.

## Supplemental Material

Supplemental Material - Challenges navigating publicly funded home care in Ontario, Canada: Perspectives from unpaid caregivers of persons living with dementia Click here for additional data file. Supplemental Material for Challenges navigating publicly funded home care in Ontario, Canada: Perspectives from unpaid caregivers of persons living with dementia by Husayn Marani, James Shaw, and Gregory P Marchildon in Dementia

## References

[bibr1-14713012231190579] Bhatia V. Orsini M. (2014). Narrating sustainability in canadian health care reform discourse. Social Policy and Administration, 50(3), 297–315. 10.1111/spol.12103.

[bibr2-14713012231190579] Canadian Institute for Health Information [CIHI] . (2020). 1 in 3 unpaid caregivers in Canada are distressed. Canadian Institute for Health Information [CIHI]. https://www.cihi.ca/en/1-in-3-unpaid-caregivers-in-canada-are-distressed

[bibr3-14713012231190579] Casey L. (2021, October 31). Ontario home care sector reports mass exodus of healthcare workers moving to hospitals, long-term care homes. CP24. https://www.cp24.com/news/ontario-home-care-sector-reports-mass-exodus-of-healthcare-workers-moving-to-hospitals-long-term-care-homes-1.5645912?cache=fbvfbdkpstrm%3FclipId%3D104066

[bibr4-14713012231190579] Chambers L. W. Bancej C. McDowell I. (2016). Prevalence and monetary costs of dementia in Canada. Alzheimer Society of Canada. https://web.archive.org/web/20180423221615id_/http://alzheimer.ca/sites/default/files/Files/national/Statistics/PrevalenceandCostsofDementia_EN.pdf

[bibr5-14713012231190579] Cheff R. (2018). Compensating research participants: A survey of current practices in toronto. https://www.wellesleyinstitute.com/wp-content/uploads/2018/07/Fair-compensation-Report-.pdf

[bibr6-14713012231190579] Cohen M. Z. Kahn D. L. Steeves D. L. (2000). Hermeneutic phenomenological research: A practical guide for nurse researchers. Sage.

[bibr7-14713012231190579] Connecting Care Act, c. 5, Sched. 1 (2019). https://www.ontario.ca/laws/statute/19c05

[bibr8-14713012231190579] Connelly J. (2001). Critical realism and health promotion: Effective practice needs an effective theory. Health Education Research, 16(2), 115–120. 10.1093/her/16.2.115. 11345656

[bibr9-14713012231190579] DaSilva L. (2015. September 23). Voices: Family caregivers need better PSW support to keep loved ones with dementia at home. Etobicoke Guardian. https://www.toronto.com/community-story/5923427-voices-family-caregivers-need-better-psw-support-to-keep-loved-ones-with-dementia-at-home/

[bibr10-14713012231190579] Flood C. M. Dejean D. Doetter L. F. Quesnel-Vallée A. Schut E. (2021. April 7). Assessing cash-for-care benefits to support aging at home in Canada. Institute for Research on Public Policy [IRPP] https://irpp.org/research-studies/assessing-cash-for-care-benefits-to-support-aging-at-home-in-canada/

[bibr11-14713012231190579] Garnett A. Browne G. (2016). The relationship between traumatic injury in children and long-term use of health and social services by children and their families. Journal of Trauma Nursing: The Official Journal of the Society of Trauma Nurses, 23(4), 215–226. 10.1097/JTN.0000000000000219. 27414144

[bibr12-14713012231190579] Genereaux D. van Karnebeek C. D. Birch P. H. (2015). Costs of caring for children with an intellectual developmental disorder. Disability and Health Journal, 8(4), 646–651. 10.1016/j.dhjo.2015.03.011. 25991418

[bibr13-14713012231190579] Gentles S. J. Charles C. Ploeg J. McKibbon K. A. (2015). Sampling in qualitative research: Insights from an overview of the methods literature. Qualitative Report, 20(11), 1772–1789. 10.46743/2160-3715/2015.2373.

[bibr14-14713012231190579] Gilson L. (Ed.), (2012). Health policy and systems research: A methodology reader alliance for health policy and systems research. World Health Organization. https://apps.who.int/iris/handle/10665/44803

[bibr15-14713012231190579] Government of Ontario . (2010). Aging at home strategy. Government of Ontario. https://news.ontario.ca/mohltc/en/2010/08/aging-at-home-strategy.html

[bibr16-14713012231190579] Government of Ontario . (2016). Developing ontario's dementia strategy: A discussion paper. Ministry of Health and Long-Term Care. https://files.ontario.ca/developing_ontarios_dementia_strategy_-_a_discussion_paper_2016-09-21.pdf

[bibr17-14713012231190579] Government of Ontario . (2022a). Connecting care act, 187. Government of Ontario. https://www.ontario.ca/laws/regulation/220187#BK1

[bibr18-14713012231190579] Government of Ontario . (2022b). Home and community care. Government of Ontario. https://www.ontario.ca/page/home-community-care

[bibr19-14713012231190579] Hapsari A. P. Ho J. W. Meaney C. Avery L. Hassen N. Jetha A. Lay M. Rotondo M. Zuberi D. Pinto A. (2022). The working conditions for personal support workers in the greater Toronto area during the COVID-19 pandemic: A mixed-methods study. Canadian Journal of Public Health, 113(6), 817–833. 10.17269/s41997-022-00643-7. 35616873 PMC9134716

[bibr20-14713012231190579] Kelly C. Jamal A. Aubrecht K. Grenier A. (2021). Emergent issues in directly-funded care: Canadian perspectives. Journal of Aging and Social Policy, 33(6), 626–646. 10.1080/08959420.2020.1745736. 32321374

[bibr21-14713012231190579] Larkin M. Watts S. Clifton E. (2006). Giving voice and making sense in interpretive phenomenological analysis. Qualitative Research in Psychology, 3(2), 102–120. 10.1191/1478088706qp062oa.

[bibr22-14713012231190579] Laucius J. (2018. July 13). 'We are in crisis': Personal support workers are the backbone of home care in Ontario — and there aren't enough of them. Ottawa, ON. https://ottawacitizen.com/news/local-news/when-the-backbone-is-broken

[bibr23-14713012231190579] Lilly M. B. Robinson C. A. Holtzman S. Bottorff J. L. ( (2012). Can we move beyond burden and burnout to support the health and wellness of family caregivers to persons with dementia? Evidence from British columbia. Health and Social Care in the Community, 20(1), 103–112. 10.1111/j.1365-2524.2011.01025.x. 21851447

[bibr24-14713012231190579] Lindeza P. Virgolino A. Santos O. Guerreiro M. Rosa M. (2020). Facing costs with dementia: Daily lives perspectives from informal caregivers (preprint). BMC Geriatrics, 10.21203/rs.3.rs-109248/v1.

[bibr25-14713012231190579] Marani H. Allin S. Marchildon G. (2021b). Development of a web-based survey on the financial risks of unpaid caregiving: Approach and lessons learned from a Canadian perspective. Home Health Care Services Quarterly, 40(4), 276–301. 10.1080/01621424.2021.1976344. 34581238

[bibr26-14713012231190579] Marani H. Allin S. McKay S. Marchildon G. (2023). The financial risks of unpaid caregiving during the COVID-19 pandemic: Results from a self-reported survey in a Canadian jurisdiction. Health Services Insights, 16, 11786329221144889. 10.1177/11786329221144889. 36643938 PMC9827143

[bibr27-14713012231190579] Marani H. McKay S. Marchildon G. (2021a). The impact of COVID-19 on the organization of personal support work in Ontario, Canada. Journal of Long-Term Care, 283–293. 10.31389/jltc.70.

[bibr28-14713012231190579] McGregor M. J. Ronald L. A. (2011, January 24). Residential long-term care for Canadian seniors: Nonprofit, for-profit or does it matter? (Study No. 14). Institute for Research on Public Policy [IRPP]. http://irpp.org/research-studies/study-no14/

[bibr29-14713012231190579] McKee M. Balabanova D. Basu S. Ricciardi W. Stuckler D. (2013). Universal health coverage: A quest for all countries but under threat in some. Value in Health: The Journal of the International Society for Pharmacoeconomics and Outcomes Research, 16(1 Suppl), S39–S45. 10.1016/j.jval.2012.10.001. 23317643

[bibr30-14713012231190579] Ontario Caregiver Organization [OCO] . (2020). Spotlight on Ontario’s caregivers. Ontario Caregiver Organization [OCO]. https://ontariocaregiver.ca/wp-content/uploads/2020/12/OCO-Spotlight-report-English-Dec10.pdf

[bibr31-14713012231190579] Olsen G. M. (1994). Locating the Canadian welfare state: Family policy and health care in Canada, Sweden, and the United States. The Canadian Journal of Sociology, 19(1), 1–20. 10.2307/3341235.

[bibr32-14713012231190579] Ontario Caregiver Organization [OCO] . (2021). Spotlight 2021 – the impact of covid-19 on caregivers: Year two. Ontario Caregiver Organization [OCO]. https://ontariocaregiver.ca/publications/oco-spotlight-report/

[bibr33-14713012231190579] Polit D. F. Beck C. T. (2010). Generalization in quantitative and qualitative research: Myths and strategies. International Journal of Nursing Studies, 47(11), 1451–1458. 10.1016/j.ijnurstu.2010.06.004. 20598692

[bibr34-14713012231190579] Sandelowski M. (1995). Qualitative analysis: What it is and how to begin. Research in Nursing and Health, 18(4), 371–375. 10.1002/nur.4770180411. 7624531

[bibr35-14713012231190579] Saunders B. Sim J. Kingstone T. Baker S. Waterfield J. Bartlam B. Burroughs H. Jinks C. (2018). Saturation in qualitative research: Exploring its conceptualization and operationalization. Quality and Quantity, 52(4), 1893–1907. 10.1007/s11135-017-0574-8. 29937585 PMC5993836

[bibr36-14713012231190579] Shaw J. A. Connelly D. M. (2012). Phenomenology and physiotherapy: Meaning in research and practice. Physical Therapy Reviews, 17(6), 398–408. 10.1179/1743288X12Y.0000000043.

[bibr37-14713012231190579] Smetanin P. Kobak P. Briante C. Stiff D. Sherman G. Ahmad S. (2009). Rising tide: The impact of dementia in Canada 2008 to 2038. Canadian Centre for Economic Analysis https://www.economic-analysis.ca/sites/economic-analysis.ca/files/reports/Rising_Tide_-_Dec_8_%28rev%29.pdf

[bibr38-14713012231190579] Spicker P. (2013). Liberal welfare states. In Greve B. (Ed.), The routledge handbook of the welfare state. Routledge (pp. 193–201). 10.4324/9781315207049.

[bibr39-14713012231190579] Taylor M. G. Quesnel-Vallée A. (2017). The structural burden of caregiving: Shared challenges in the United States and Canada. The Gerontologist, 57(1), 19–25. 10.1093/gerontol/gnw102. 27521577

[bibr40-14713012231190579] van Manen M. Higgins I. van der Riet P. (2016). A conversation with Max van Manen on phenomenology in its original sense. Nursing and Health Sciences, 18(1), 4–7. 10.1111/nhs.12274. 26931367

[bibr41-14713012231190579] Zagrodney K. A. P. Bolongaita L. McKay S. M. Nichol K. King E. C. (2021). Examining the effect of COVID-19 policies on personal support worker (PSW) leaves of absence in a Canadian home care setting. IHEA. [Poster presentation]. https://www.vha.ca/wp-content/uploads/2021/07/Research-Project-LOA-iHEA-EffectCOVIDPoliciesLeavesAbsencePSW.pdf

