# Supplementary material for: Challenges navigating publicly funded home care in Ontario, Canada: Perspectives from unpaid caregivers of persons living with dementia
Source: Dementia (London). 2023 Jul 19;22(7):1626–45. doi: 10.1177/14713012231190579 (PMC10521154; doi:10.1177/14713012231190579)
Supplement: Supplemental Material - Challenges navigating publicly funded home care in Ontario, Canada: Perspectives from unpaid caregivers of persons living with dementia [file sj-pdf-1-dem-10.1177_14713012231190579.pdf]

Supplementary Table 1 Semi-Structured Interview Guide

Topic Area	Interview Question(s)	Prompts
1. General caregiving roles (interpretation of what caregiving means/entails)	Tell me about your day-to-day life as a caregiver.<br><br>Why did you assume this role as family caregiver?	E.g., years of caregiving experience; full-time or part-time caregiving status; shared caregiving responsibilities (e.g., with sibling or hired help); live-in or commuter caregiver; stage of dementia family member living with dementia is at (and any existing co-morbidities they have); prior care family member living with dementia was in (e.g., long-term care); duties as caregiver (bathing, cooking, feeding, taking to appointments, etc.).
2. General interpretations of own well-being as a caregiver. General understanding of their own interpretation of the impact of caring (not necessarily financial impact, e.g., productivity, health status, quality of life).	Describe some of the best and worst aspects of caregiving. Where do you think you fall on this continuum between best and worst?<br><br>In terms of your employment, the number of hours you work stayed the same, increased or decreased since you began caregiving?	*If stressed, unhealthy, depressed, etc.:* What do you think has contributed to these feelings?<br><br>*If happy, content, well:* What do you think has contributed to these feelings?
3. Understanding caregiver expenses within the home and as a result of caring.	Tell me about what you had to do to prepare for caregiving.<br><br>Tell me about some of your day-to-day expenses.<br><br>Can you give me an estimate of how much you pay for certain	Did you have to stop working full-time? Did you reduce your hours at work? Have you had to renovate the home of your (relative living with dementia)? Did you seek assistance from a personal care worker? Are you or your dependent on any prescription medications? How often do you visit a health care provider with your

	care expenses?<br><br>How do you pay for various expenses (e.g., medications)?	dependent—do you drive them there? How many times per day do you eat? Are there any important food or diet-related considerations for your dependent? Do you cook for your dependent? You mentioned you feel tired and stressed, have you heard of respite options for your own well-being? Do you participate in caregiver respite programs?<br><br>What are some of your most costly expenses? Are any of these expenses reimbursed? Do you share these expenses (caregiving or otherwise) with anyone else? How have these costs changed over the progression of your relative's dementia? Are these costs restricted to dementia only, or your dependent's other illnesses?
4. Interpretation of these expenses	Before you started caregiving, have your overall expenses gone up, down, or stayed the same?	Tell me about some of the personal expenses you have forgone, if any. Do you ever limit personal spending (leisure/vacation, family, personal health expenses)? Since you stated you are trying to save money, describe some strategies that you have taken to save money in your day-to-day life?
	Compared to before you started caregiving, are you better off or worse off or the same financially?<br><br>Describe your ability to meet the financial expectations of caregiving.	How does this make you feel? E.g., worried? indifferent?<br><br>Do you feel like you can do "this" (assume financial responsibility of care) on your own, or not? If so, fully or inadequately?<br><br>Describe the emotions you experience in terms of the costs of caring (are you happy, stressed, it depends on the day?) How often do

		you spend on yourself? Do you go on vacation?
5. Financial information, financial means, financial support, and financial risk protection	*Financial information:*<br><br>Where did you receive your information around costs of caring?<br><br>How would you assess your financial literacy in this area?	Do you/did you receive your information around how much things cost, and if so, from where: internet, your pharmacist, word-of-mouth, etc.? Was there a learning curve at first where you needed access to information on how much certain things costs, but you did not have this access? If you have a PSW, how did you find them?
	Financial means:<br><br>Is your income ever a concern for you? How does this make you feel?	How much of your own income goes toward covering caregiving costs for caring for your dependent relative?<br><br>Do you have any other sources of income? Do you have any savings? Currently or in the past, have you had to take on additional paid work, borrow money, or supplement your income through other means?
	Financial support:<br><br>Do you receive any financial support at all?<br><br>Did you have to leave your job when you began caregiving, or reduce your hours? How did your current or former employer support you in terms of care benefits and/or a leave of absence?	*If yes*, from whom (e.g., government or other people)? How does this financial support help you (e.g., is this support helping you to offset your previously identified care or personal costs)? Do you need more support? If so, how could the supports you're currently receiving be improved? What are your expectations of public financial support?<br><br>*If no, why?* Have you heard of government financial support schemes?

	Process Issues:<br><br>How would you describe accessing caregiver supports such as financial support?<br><br>How do you see these programs in terms of helping or benefitting you?<br><br>How did you feel filling out any forms that asked you to share your income?	Are programs accessible to you? Are you able to access multiple types of programs or do you face any restrictions? Do you have to demonstrate or prove that you need the support or that your recipient of care has a condition? How rigid are the steps? If you miss a step are you ineligible? Do you have to wait to be reimbursed? How do you feel asking for money? Do you receive help filling out forms? How do you fill out forms – online or on paper?
6. Impact of caregiver costs on other aspects of life	Describe what effective "caregiver support" should look like for others in your position.	Do you have any advice for other caregivers around the costs of care?

Supplementary Table 2 Codebook

**Care Activities**
Activities that constitute care
Bathing
Counselling and easing anxieties
Financial management (of care recipient's account)
Banking
Preparing a will
Provides care for another dependent relative (e.g., parent, spouse, child)
Same things PSWs do
Challenges in performing care activities
Constant supervision needed
Impact: Compromised personal leisure
Impact: Compromised respite
Impact: Compromises ability to work
Request for additional services denied
COVID impacted service availability
COVID preventing certain activities
House limitations (physical limitations of structure of home)
Specific help needs or support required based on nature of dementia (e.g., respite, 24/7 care, discomforts)
Unpredictability of dementia challenging
Sharing care activities with someone
Care activities shared with parent
Care activities shared with spouse
**Care Expenses**
Foregone expenses (recreation, leisure, food)
COVID has helped to save money

Impact of COVID on expenses
Characterizing care expenses
Care expenses are shared
Care expenses publicly subsidized (e.g., PSWs, bed)
Day program (hours and cost)
Equipment (e.g., bed)
Footcare
Groceries
Medications
Physician forms
Home care workers (e.g., personal support workers)
Renovations
Unforeseen
Perspectives on expenses
Change in care expenses over dementia trajectory (increased)
Does not talk about finances
Have to pay if forced to (no other alternative)
Indirect costs more significant (e.g., time away from work, mental distress)
Taking it month-by-month
Always choose the cheapest option of expenses are paid out-of-pocket
**Caregiver Health**
Care exacerbating existing health challenges
COVID compromised personal health provision
Impact on mental health
Challenges in getting help for mental health
COVID exacerbated (or benefited) mental health
Desire for respite or a break
Expenses for personal mental health
Impact on physical health
**Defining "caregiving"**
"Not sure how long I can do this"
Likening dementia care to childcare
Perceptions of care and labour
Extent to which care should be compensated
Who should compensate
Prepared to care (previous experience)
**Employment and Care**
Balancing employment and care
Delayed retirement
Employer support (yes or no)
Desired support from employer
Impact on applying for jobs
Retired early
Taking time off work (voluntarily or involuntarily)
Distracts from productivity at work (constant communication, missing deadlines)
**Income**
Impacts of care on income level
Income sources now (savings, pension, investments)
Income sources (in)sufficient
Preparedness to meet care expenses (savings)
Supplementing income to pay expenses
**Information and related-needs**
Questions about care costs or requests for information deferred elsewhere

Perspectives on responsibility for the dissemination of care info (e.g., costs)
Sources of information (financing, dementia, caring, etc.)
Accountant or financial planner
Alzheimer Society (national or local chapter)
Home and Community Care Support Services (HCCSS), formerly Local Health Integration Network (LHIN)
Pension office
Physician (specialty or general)
Word-of-mouth or social media
**Notions of home care (vs. facility-based long-term care)**
Cognizant or caregiver said no to being put in residential long-term care facility
Negative perception of long-term care (e.g., expensive, waitlist)
Private congregate home is expensive
Would not put care recipient in facility-based long-term care (e.g., because of personal relationship)
Preference of facility-based long-term care
Awaiting placement (on wait-list or delayed because of COVID-19)
Socialization is a benefit of living in long-term care facility
**Personal support workers**
Hours of paid work (sufficient vs. insufficient)
Filling in due to inadequate/inconsistent PSWs (e.g., hours)
LHIN dependent on caregivers to fill gaps
Willingness to pay out-of-pocket for additional hours
COVID-19 impacted availability of PSWs
Belief that PSWs need to be better trained in dementia care
PSWs derived from multiple sources (e.g., LHIN, privately)
Lack of continuity
PSWs offer caregivers respite, but not always
**Profile of care recipient**
Co-morbidities exacerbated by dementia
COVID-19 has aggravated symptoms
COVID-19 has compromised socialization
Living arrangement (e.g., formerly living independently)
General personality (e.g., resistant to PSWs due to personality)
Surrendered license
Sleep schedule (creates challenges in caregiving)
Stage of illness (changes in symptoms and impact on caregiving)
Type of Dementia
**Public support to manage financial risks of caregiving**
Perspectives on financial support
Design and considerations (e.g., eligibility must be equitable)
Types of care expenses that should be subsidized
Extent to which public (government) should compensate caregivers
Yes: Government should be partners in care
Yes: Caregivers should be salaried/paid to stay at home
Yes: Government wants people in their own homes, so caregivers should be incentivized
Yes: Caregivers are paying taxes and should therefore be paid to provide care
No: Financially secure
No: Do not need to be rewarded for something I feel obligated to do/would do for free (out of love)
No or few public supports
Because care recipient does not want it
Because caregiver does not consider themselves as needing it
Because of eligibility restrictions by government (e.g., LHIN, federal government)
Because they were completely denied by public sources (e.g., government)
Because they never applied or were unaware

Extent of support from others to meet care expenses (e.g., family)
Sources of support (e.g., financial support, services, assistive devices)
Alzheimer Society
City of Toronto
Government (e.g., Canada Pension Plan, Disability Tax Credit, Employment Insurance, Ontario Works)
HCCSS or LHIN
Interaction with case managers
March of Dimes
Service Provider Organizations
Veterans Affairs Canada
